# Metabolism modulation in rat tissues in response to point specificity of electroacupuncture

**DOI:** 10.1038/s41598-021-04382-6

**Published:** 2022-01-07

**Authors:** Der-Yen Lee, Yu-Rung Jiu, Ching-Liang Hsieh

**Affiliations:** 1 Graduate Institute of Integrated Medicine, College of Chinese Medicine, China Medical University, Taichung, 40402 Taiwan; 2 School of Chinese Medicine, College of Chinese Medicine, China Medical University, Taichung, 40402 Taiwan; 3 Chinese Medicine Research Center, China Medical University, Taichung, 40402 Taiwan; 4 Graduate Institute of Acupuncture Science, College of Chinese Medicine, China Medical University, 91 Hsueh-Shih Road, Taichung, 40402 Taiwan; 5 Department of Chinese Medicine, China Medical University Hospital, Taichung, 40447 Taiwan

**Keywords:** Metabolomics, Analytical biochemistry, Biological models

## Abstract

Zusanli (ST36) and Neiguan (PC6) are acupoints along two meridians. To demonstrate point specificity, we investigated the effects of ST36 and PC6 in electroacupuncture (EA)-treated rats. The rats were subjected to sham acupuncture at ST36 without electric stimulation, EA at ST36, or EA at PC6. Heart and stomach tissues were collected for metabolite profiling. Each type of stimulation resulted in a different metabolite composition in the rat heart and stomach tissues. In the heart tissues, EA at ST36 affected a wider range of metabolite pathways than did EA at PC6, whereas similar numbers of metabolites in the stomach tissues were affected by EA at ST36 and PC6. The pathways affected by EA at ST36 differed from those affected by EA at PC6, and a group of common metabolites were reversely regulated by these two acupoints. This study demonstrated point specificity effectively modulated metabolism in rat heart and stomach tissues. The results indicate that heart stimulation may be connected to the stomach through the pericardium meridian (as described in traditional Chinese medicine), explaining why acupuncture applied to the stomach meridian can be an alternative treatment for gastric and heart diseases.

## Introduction

The meridian system and acupoints are crucial to treating disease in acupuncture. In traditional Chinese medicine (TCM), the meridian system refers to the channels for the circulation of qi, and blood connects acupoints and internal organs. Each acupoint corresponds to an internal organ; for example, the Zusanli acupoint (ST36) is the lower He-sea acupoint on the stomach meridian, and acupuncture signals are transmitted via ST36 to the stomach. Similarly, acupuncture signals are transmitted via the Neiguan acupoint (PC6) to the heart. This concept is called “acupoint specificity.” Our previous study demonstrated that 2-Hz electroacupuncture (EA) applied to ST36 and PC6 can affect neurotransmitter levels in multiple areas of the brain^1^. Both the stomach and heart are closely related to energy generation and metabolism, and acupuncture at ST36 can induce multiple metabolic pathways in serum, including glycerophospholipid and fatty acid metabolism^2,3^. We investigated whether EA at ST36 and PC6 also affects the metabolism of these acupoints’ corresponding target internal organs.

Acupuncture can be applied to PC6 to regulate cardiovascular function by inhibiting the sympathoexcitatory cardiovascular reflex response via a long-loop pathway of the hypothalamic rostral ventrolateral medulla, arcuate nucleus, and ventrolateral periaqueductal gray. In addition, γ-aminobutyric acid, an opioid, and 5-hydroxytryptamine are involved^4^. Acupuncture at PC6 can increase left ventricular diastolic and systolic function in an isoproterenol-induced myocardial ischemia rat model; this suggests that acupuncture at PC6 can affect cardiac function^5^. Acupuncture at PC6 is more effective than acupuncture at ST36 for treating chronic myocardial ischemia in minipigs in an amberoid constrictor model^6^. Acupuncture at ST36 with transcranial magnetic stimulation can increase motor cortical excitation and reduce motor cortical inhibition^7^. EA applied bilaterally to ST36 and the Xiajuxu acupoint for 60 min can trigger an increase in extracellular Ca^2+^, K^+^, Na^+^, and Cl^-^ levels to increase the uptake of gastric wall Na^99m^TcO_4_^8^. Low- and high-frequency EA at ST36 can restore a damaged network of Cajal interstitial cells, thereby increasing the gastric emptying time of streptozotocin-induced diabetic rats^9^. EA at ST36 can further the reduction of antral motility and gastric slow waves in dogs with rectal distension through vagal activity and partial opioid pathways^10^. Therefore, acupuncture at PC6 and ST36 can influence heart and stomach function, respectively, through separate pathways.

ST36 can be used to treat cardiovascular disorders such as precordial pain and palpitation, whereas PC6 can be used to treat digestive disorders such as vomiting and stomachache^11,12^. Our previous metabolomic analysis revealed that EA at PC6 and ST36 induces multiple neurotransmitter profiles in the cerebral cortex and hippocampus of rats^1^. Therefore, in this study, we performed metabolite profiling to determine the effects of EA at PC6 and ST36 on the metabolism of rat heart and stomach tissues.

## Results

### Investigating point specificity by analyzing metabolite composition in rat tissues

**Figure 1:**
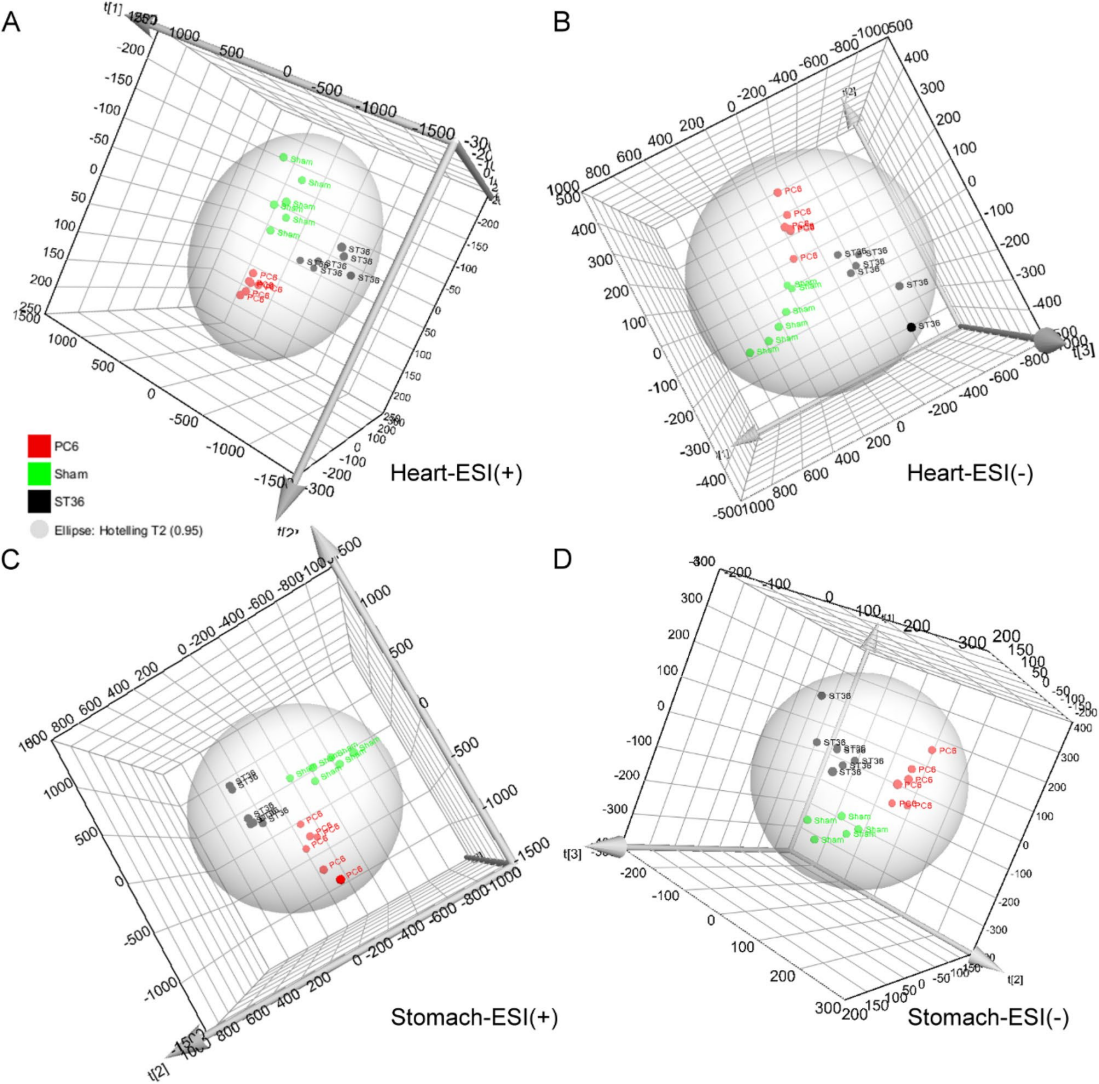
Score plots of effects of EA treatment on metabolite signals in rat heart and stomach tissues. PLS-DA of (**A**) LC–ESI(+)–MS and (**B**) LC–ESI(−)–MS signals in the heart tissues and (**C**) LC–ESI(+)–MS and (**D**) LC–ESI(−)–MS signals in the stomach tissues after EA at ST36, EA at PC6, and sham treatment. Input signals for PLS-DA were collected with the criteria of fold change > 2, *p* < 0.05, and CV (coefficient of variation) < 30 by comparing the three types of EA in Progenesis QI with EZinfo.

Heart and stomach tissues from EA-treated rats were subjected to sample preparation for metabolite profiling. Tissue metabolite extracts were then resolved through liquid chromatography-electrospray ionization-mass spectrometry (LC-SEI-MS) to acquire metabolite data. LC–MS signals were processed using partial least squares discriminant analysis (PLS-DA) to generate score plots; the processed LC–MS signals were acquired using positive (Fig. 1A) and negative (Fig. 1B) modes for heart tissues and positive (Fig. 1C) and negative (Fig. 1D) modes for stomach tissues. The plots indicated that the data points for a given EA treatment were close together and grouped by type and location of treatment.

### Potential to influence metabolites in rat tissues through acupoints

**Figure 2:**
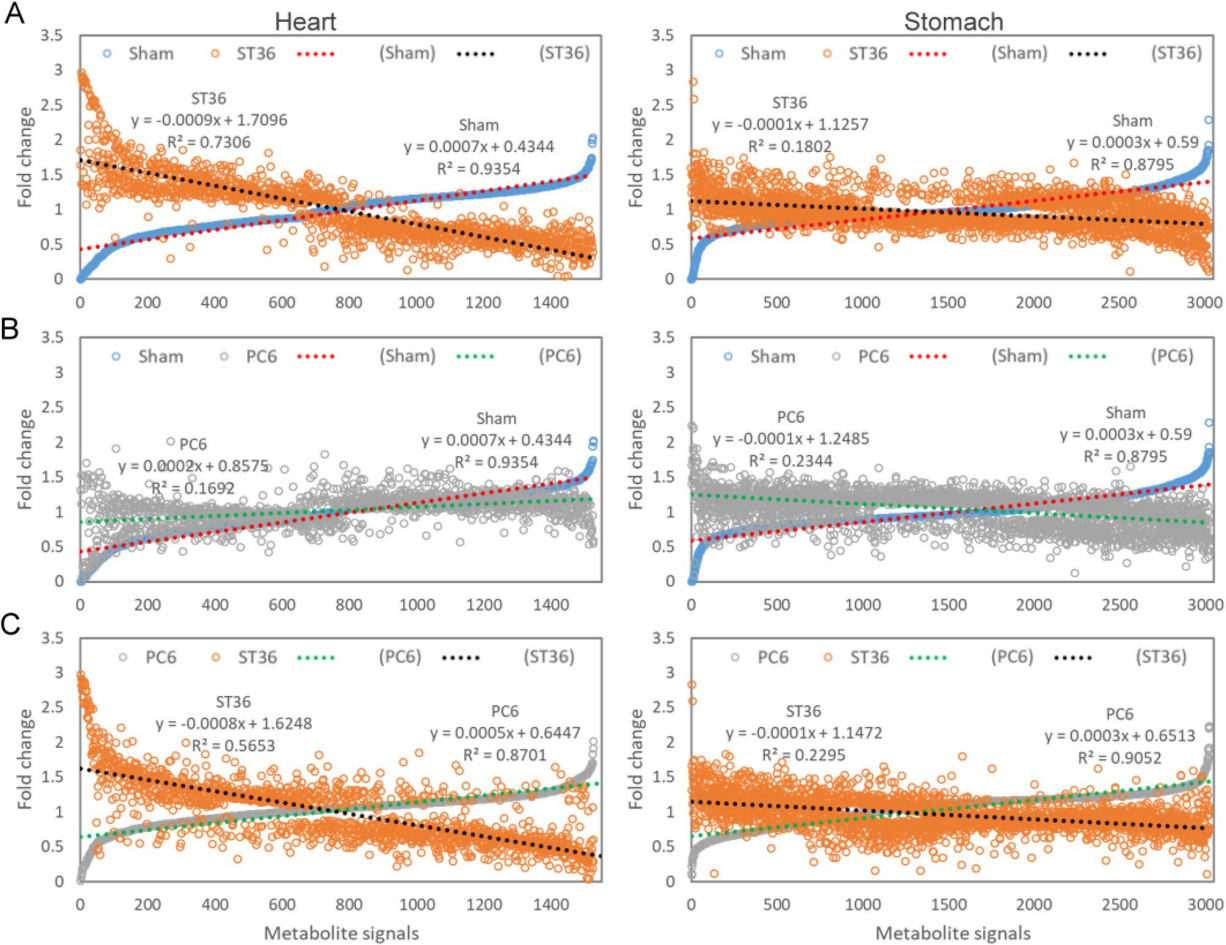
Scatter diagrams for fold change in metabolite signals in heart and stomach tissues of EA-treated rats. Each fold change in the metabolite signals was calculated by dividing the average number of LC–MS signals observed for each EA treatment by the average for all samples (*n* = 6 for each EA treatment; total *n* = 18). The fold changes in the metabolite signals in the heart and stomach tissues of the EA-treated rats were analyzed by combining (**A**) the ST36 stimulation and sham treatment, (**B**) the PC6 stimulation and sham treatment, and (**C**) the ST36 and PC6 stimulation. The results were combined with the detected LC–MS signals in the ESI + and ESI − modes.

To determine the effects of applying EA (to ST36 or PC6) or sham treatment to metabolites in rat heart and stomach tissues, we used scatter diagrams to compare the fold change of each metabolite signal obtained from PLS-DA. After the fold change values of the sham group dataset were arranged in ascending order, we discovered that the fold changes of the correlated signals in the ST36 group dataset represented a reverse trend, with a gap between the two datasets. For the heart samples, the slopes of the ST36 and sham group datasets were − 0.0009 and 0.0007, respectively; for the stomach samples, the slopes of the ST36 and sham group datasets were − 0.0001 and 0.0003, respectively (Fig. 2A). For rat heart samples, in contrast to the results for the ST36 group, the PC6 group dataset revealed a slope of 0.0002, similar to that of the sham group dataset (Fig. 2B). However, for the stomach samples, the scatter diagrams exhibited similar data point distributions for the PC6 and ST36 groups when compared with those of the sham group (Fig. 2A,B). After the fold change values of the PC6 group dataset were arranged in ascending order, we discovered that the fold changes of the correlated signals in the ST36 group dataset also represented a reverse trend, with a gap between the two datasets. For the heart samples, the slopes of the ST36 and PC6 group datasets were − 0.0008 and 0.0005, respectively; for the stomach samples, the slopes of the ST36 and PC6 group datasets were − 0.0001 and 0.0003, respectively (Fig. 2C).

### Range of ST36 and PC6 stimulation effects on metabolites

**Figure 3:**
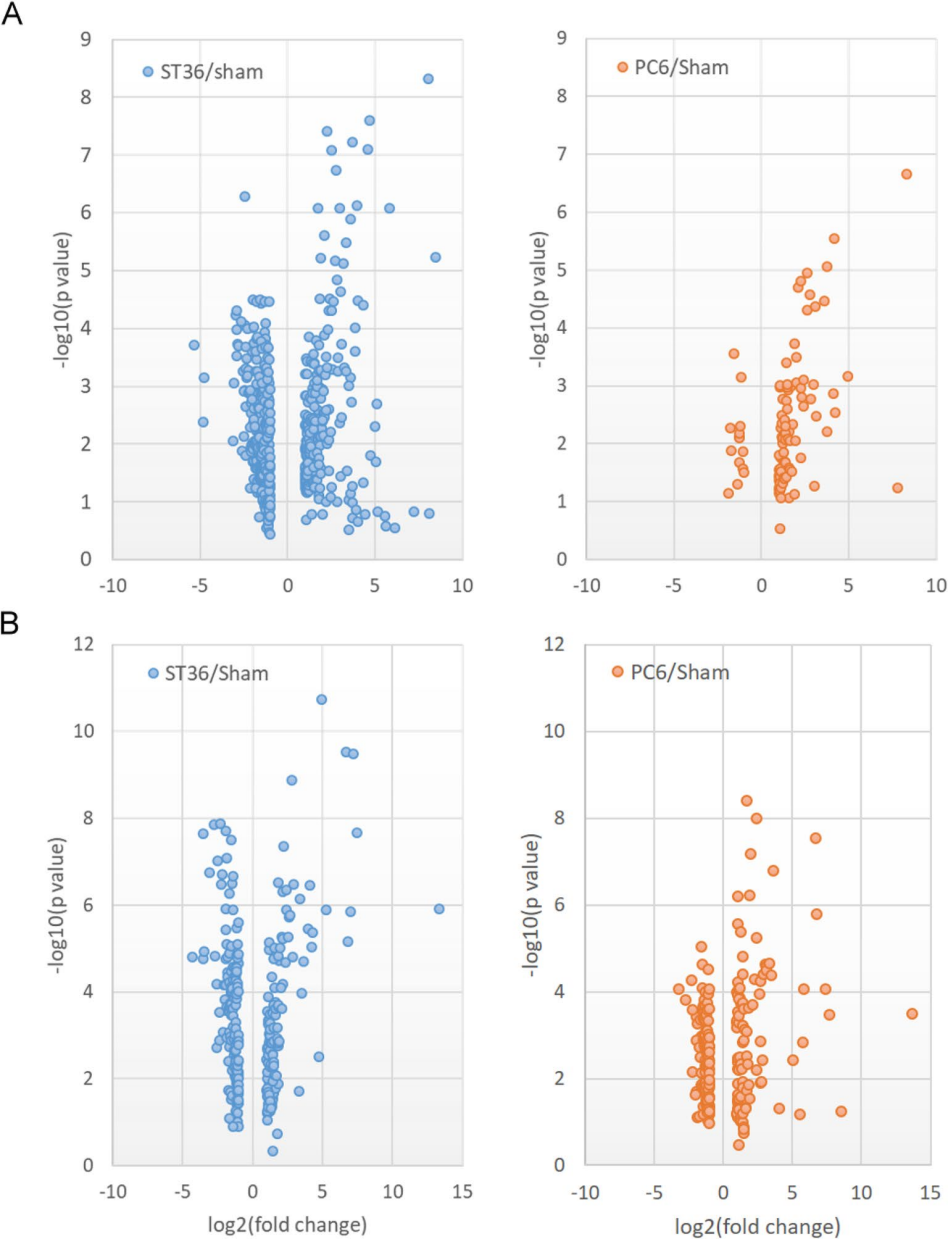
Volcano plots of metabolite signals with significant fold changes in heart and stomach tissues of EA-treated rats. Metabolite signals with fold changes (ST36/sham > 2; PC6/sham > 2) in rat (**A**) heart and (**B**) stomach tissues were plotted by log2(fold change) with − log10(*p* value). The results were summarized by filtering the detected LC–MS signals from the ESI + and ESI − modes (*n* = 6 for each EA treatment).

The ST36–sham and PC6–sham metabolite signal ratios were determined using the criteria of fold change > 2 and *p* < 0.05 to create volcano plots with a logarithmic scale. For the rat heart tissues, the number of metabolite signals with significant fold changes was 634 for ST36–sham and 99 for PC6–sham (Fig. 3A). However, for the rat stomach samples, the results and plots indicated that similar numbers of metabolite signals with significant fold changes were observed: 283 for ST36–sham and 242 for PC6–sham (Fig. 3B). The data indicate that a wider range of metabolites in the rat heart tissues was affected by EA treatment at ST36 than by EA treatment at PC6; however, in the rat stomach tissues, EA treatment at both ST36 and at PC6 induced changes to a similar range of metabolites.

### Categorizing the effects of ST36 and PC6 stimulation by identified metabolites

**Figure 4:**
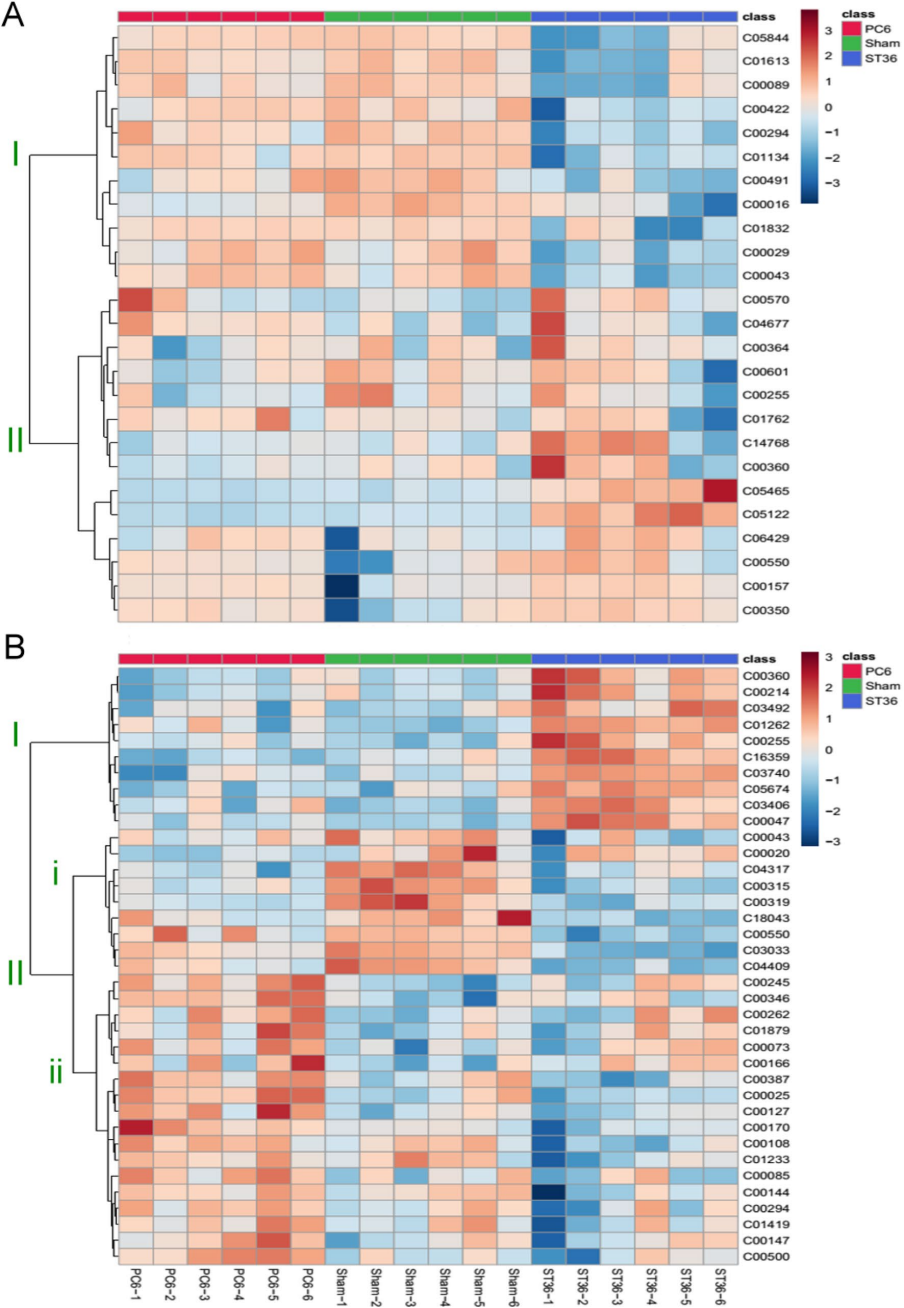
Heatmaps of identified metabolites in heart and stomach tissues of EA-treated rats. The levels of identified metabolites with KEGG ID were identified in rat (**A**) heart and (**B**) stomach tissues. The graphics were created by using MetaboAnalysis 5.0 tool (https://www.metaboanalyst.ca//faces/ModuleView.xhtml).

The LC–MS datasets were compared using the Human Metabolome Database for metabolite identification. Among the ST36, PC6, and sham groups, 25 metabolites in heart tissues and 37 metabolites in stomach tissues were identified as having undergone a significant change. The identified metabolites were analyzed using MetaboAnalysis 5.0. A heatmap of the relative metabolite levels in the rat heart tissues was plotted, and the results for the ST36, PC6, and sham groups were organized by separating the correlated metabolites into two clusters. With respect to these two clusters, the ST36 group exhibited a pattern that differed from those exhibited by the PC6 and sham groups. In the ST36 group, metabolite levels were generally upregulated in the cluster I block and downregulated in the cluster II block (Fig. 4A). The heatmap generated using rat stomach data indicated three clusters (i.e., I, II-I, and II-ii) of metabolites for the ST36, PC6, and sham groups (Fig. 4B). For the ST36, sham, and PC6 groups, metabolite levels were generally upregulated in the cluster I, cluster II-i, and cluster II-ii blocks, respectively.

**Figure 5:**
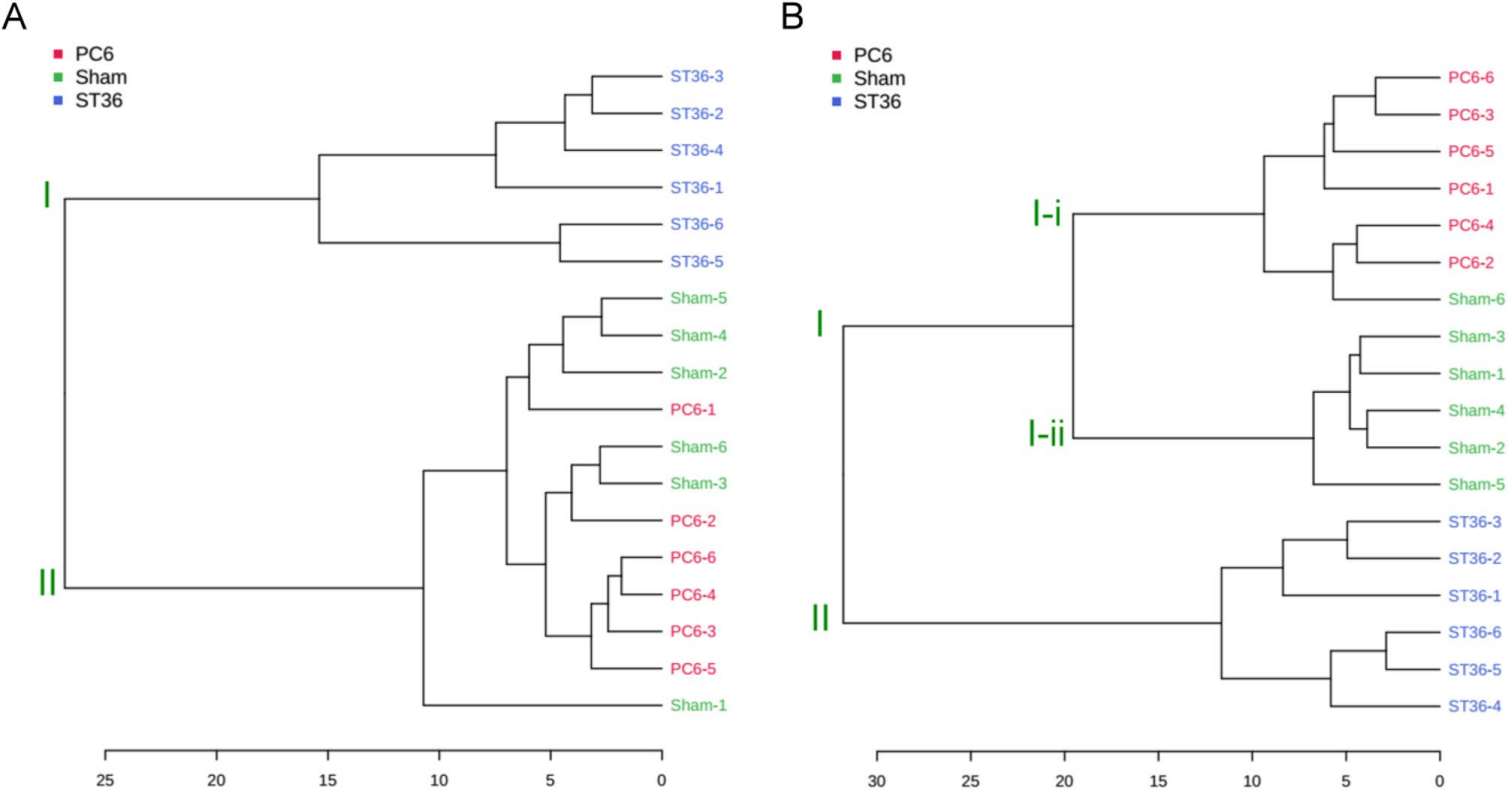
Dendrograms for effects of each EA treatment on metabolites in rat heart and stomach tissues. The clustering results for each EA treatment were interpreted by analyzing the change in identified metabolites in the (**A**) hearts or (**B**) stomachs of the EA-treated rats. The graphics were created by using MetaboAnalysis 5.0 tool (https://www.metaboanalyst.ca//faces/ModuleView.xhtml).

Figure 4 displays the levels of the identified metabolites; the correlation among the samples was determined by identifying similarities in metabolite composition. For the rat heart tissues, the samples from the ST36 group formed a main cluster I block that differed from the main cluster II blocks formed by the PC6 and sham groups (Fig. 5A). For the rat stomach tissues, the samples from the ST36 group also formed a main cluster II block that differed from the main cluster I blocks formed by the PC6 and sham groups (Fig. 5B). The samples from the PC6 and sham groups were segmented into subclusters I-i and I-ii (Fig. 5B). For the heart tissue samples from the PC6 and sham groups, the results were clustered in a similar location.

### Broad modulation of metabolism in rat heart tissues through ST36 stimulation

**Figure 6:**
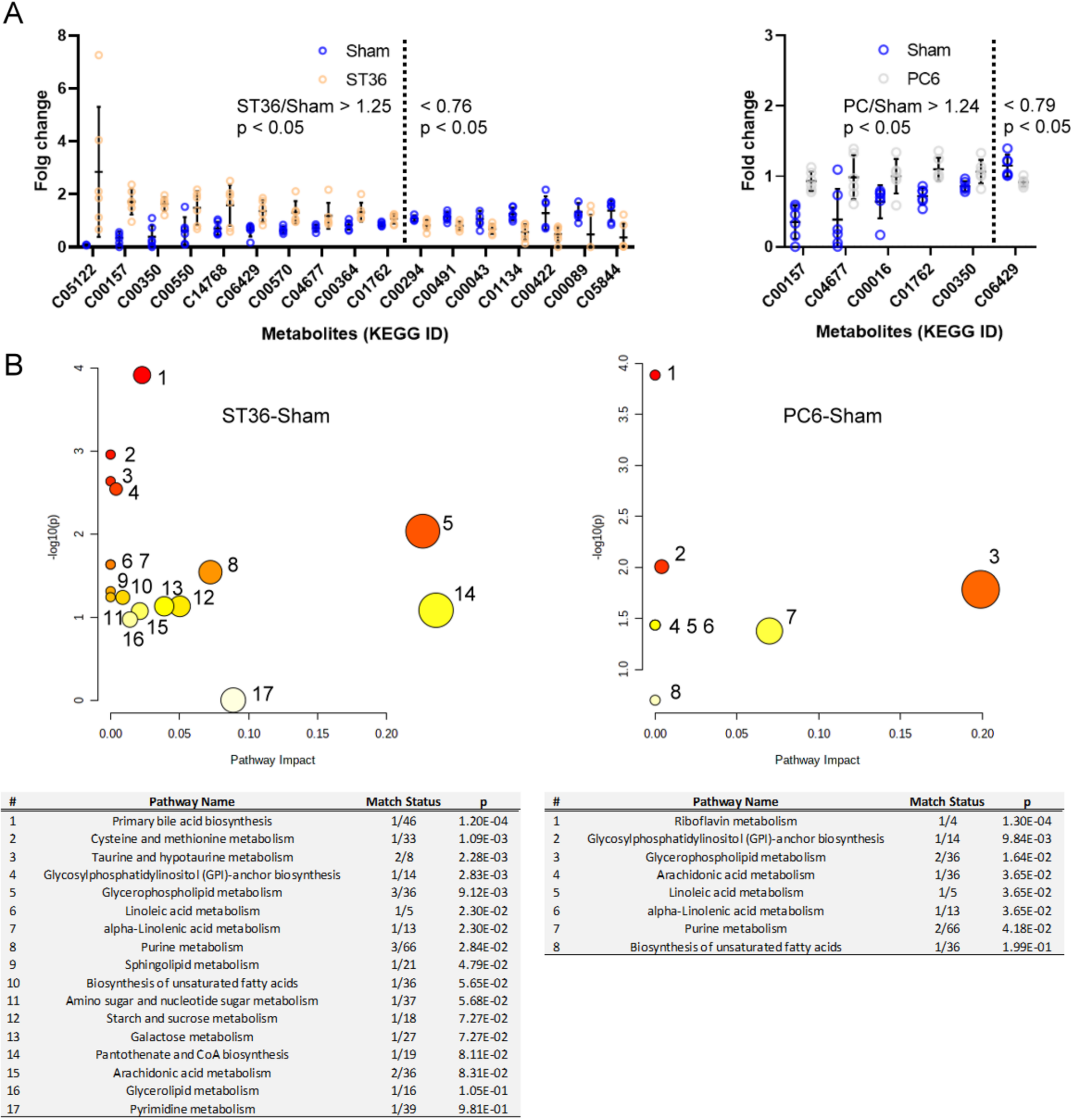
Metabolism modulated in rat hearts by each EA treatment. The (**A**) metabolites and (**B**) pathways affected by ST36 or PC6 were compared with the results of the sham treatment (*n* = 6 for each EA treatment; MetaboAnalysis 5.0 tool (https://www.metaboanalyst.ca//faces/ModuleView.xhtml)).

We examined the effects of ST36 and PC6 stimulation on metabolism in rat tissues by further investigating the metabolite levels and related pathways. By using the metabolite levels detected in the heart tissues of the sham group rats as the baseline, we discovered that EA at ST36 caused the upregulation of 10 metabolites and downregulation of 7 metabolites. However, EA at PC6 caused the upregulation of five metabolites and downregulation of one metabolite in the rat heart tissues (Fig. 6A). A pathway analysis of the data was performed using MetaboAnalysis 5.0; the analysis revealed that at least 17 and 8 metabolism pathways in the rat heart tissues were affected by EA at ST36 and PC6, respectively (Fig. 6B). The pathway involved in riboflavin metabolism was only affected by EA at PC6, and 10 pathways were only affected by EA at ST36.

### Differences in metabolism pathway modulation in rat stomach tissues between ST36 and PC6 stimulation

**Figure 7:**
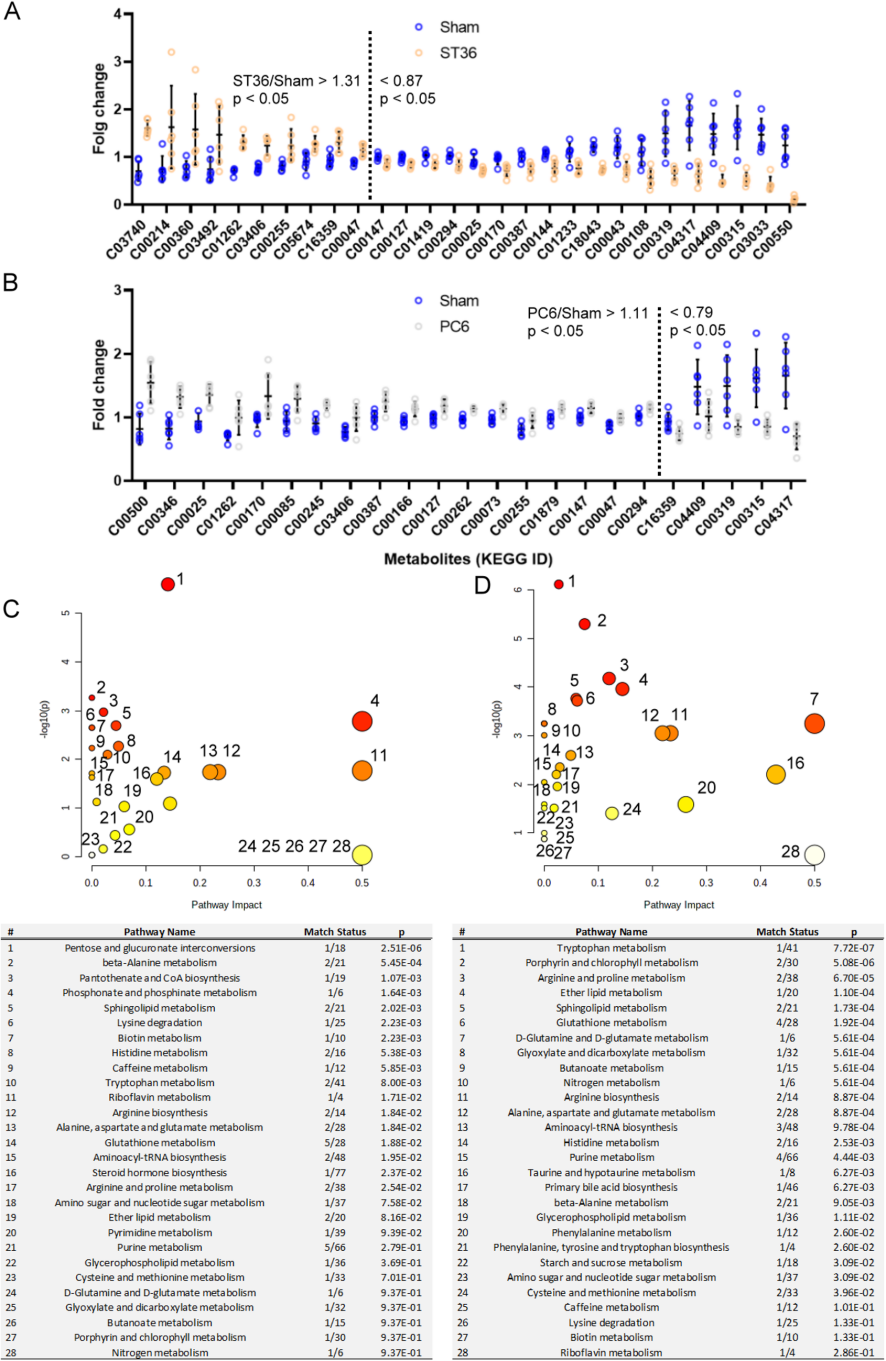
Metabolism modulated in rat stomachs by each EA treatment. The (**A**) metabolites and (**B**) pathways affected by ST36 or PC6 were compared with the results of the sham treatment (*n* = 6 for each EA treatment; MetaboAnalysis 5.0 tool (https://www.metaboanalyst.ca//faces/ModuleView.xhtml)).

The stomach tissues of the rats in the ST36 group indicated that EA at ST36 caused the upregulation and downregulation of 10 and 18 metabolites, respectively. In addition, EA at PC6 caused the upregulation and downregulation of 18 and 5 metabolites, respectively (Fig. 7A). The pathway analysis revealed that EA at ST36 and PC6 affected at least 28 metabolism pathways each in the rat stomach tissues (Fig. 7B). Only nine pathways were affected by both ST36 and PC6 stimulation. Therefore, EA at ST36 and PC6 affected metabolism in the rat stomach tissues to similar degrees but via different pathways.

### Reverse modulation of ST36 and PC6 stimulation on metabolism pathways

**Figure 8:**
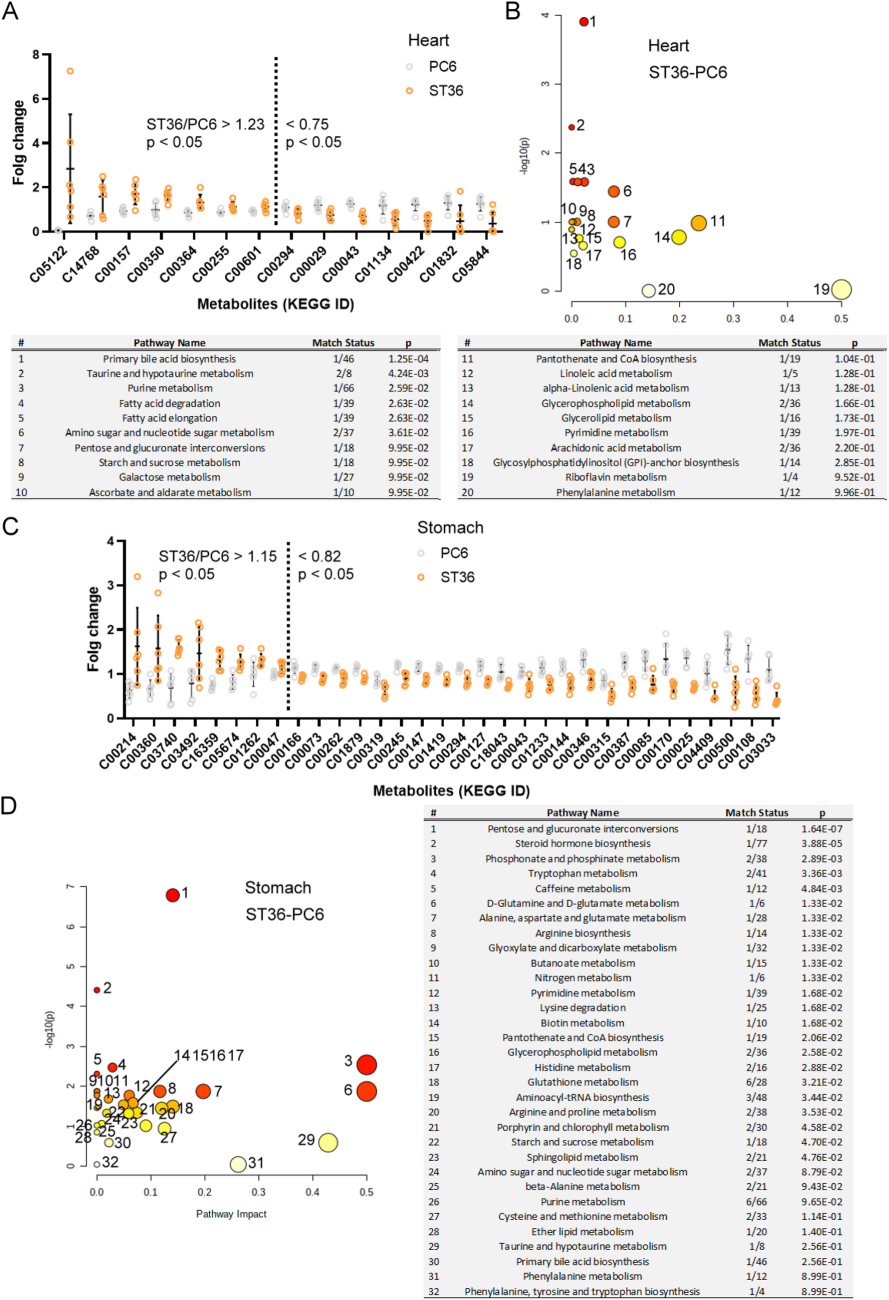
Effects of ST36 and PC6 on common metabolism pathways. (**A**) Metabolite levels and (**B**) affected pathways in the rat hearts. (**C**) Metabolite levels and (**D**) affected pathways in the rat stomachs (*n* = 6 for each EA treatment; MetaboAnalysis 5.0 tool (https://www.metaboanalyst.ca//faces/ModuleView.xhtml)).

To further evaluate the effects of ST36 and PC6 stimulation on the pathways, we identified substantial changes in the metabolite levels in the rats subjected to EA at ST36 and PC6. Compared with the metabolite levels detected in the heart tissues of the rats subjected to EA at PC6, seven metabolites were upregulated and seven metabolites were downregulated in the heart tissues of rats subjected to EA at ST36 (Fig. 8A). The pathway analysis of these metabolite changes indicated that ST36 and PC6 stimulation reversely modulated at least 20 metabolism pathways each in the rat heart tissues (Fig. 8B). In the rat stomach tissues, EA at ST36 induced the upregulation of at least 8 metabolites and downregulation of at least 24 metabolites relative to EA at PC6 (Fig. 8C). The pathway analysis indicated that ST36 and PC6 stimulation reversely modulated at least 32 metabolism pathways each in the rat stomach tissues (Fig. 8B).

## Discussion

The PLS-DA results for the LC–ESI–MS signals indicated that metabolite composition in the rat heart and stomach tissues was affected by acupuncture type (Fig. 1). These results are consistent with those regarding the effects of EA on the cerebral cortex, hippocampus, and hypothalamus from our previous study^1^; this suggests that EA-generated signals can target the heart and stomach for local modulation via the sham, ST36, and PC6 acupoints, which was also the case with EA signals and the brain. This finding is also consistent with those of other studies. The activation of multiple brain regions, including the occipital and somatosensory cortices, can be differentiated by acupuncture applied to the Guangming (GB37) acupoints and their proximal nonacupoints^13^. A functional magnetic resonance imaging study revealed that acupuncture can modulate the activity of specific regions of the brain^14^. These findings indicate that acupoint specificity was observed in the metabolic reprogramming of rat heart and stomach tissues. The results also demonstrated the manner in which the meridian system connects internal organs to the acupoints of the four extremities in TCM^15^.

The scatter diagrams revealed that for the heart tissues, the fold change of the metabolite signals in the ST36 and sham groups had an inverse relationship, whereas the trends exhibited by the PC6 and sham groups were similar. For the stomach tissues, similar patterns between the ST36 and sham groups and between the PC6 and sham groups were observed (Fig. 2A,B). In addition, the volcano plots indicated that in the heart tissues, 634 and 99 metabolite signals with significant fold changes were identified through the ST36–sham and PC6–sham comparisons, respectively; in the stomach tissues, 283 and 242 metabolite signals with significant fold changes were identified through the ST36–sham and PC6–sham comparisons, respectively (Fig. 3). The data indicate that a wider range of metabolites in the rat heart tissues was affected by ST36 stimulation than by PC6 stimulation, and similar numbers of metabolites were affected by EA at ST36 and PC6 in the rat stomach tissues (Figs. 2, 3). EA at PC6 can enhance efferent vagal activity and increase gastric motility in rats; this is consistent with our findings regarding EA at ST36^16^. In patients with rheumatic diseases accompanied by chemotherapy-induced nausea, acupuncture at PC6 combined with ondansetron can reduce the severity and duration of nausea to a greater extent than can treatment with ondansetron alone; this suggests that acupuncture at PC6 can also be used to treat gastric disease^17^. PC6 is located along the pericardial meridian connecting internal organs including the heart and stomach; in TCM, PC6 can be used to treat heart and gastric diseases^18^. The mechanism by which EA at ST36 generates a wider range of effects than does EA at PC6 is poorly understood (Figs. 2, 3). ST36 is located along the stomach meridian of Foot-Yangming, which is connected to the stomach and spleen^18^. In TCM, the spleen and stomach supply nutrients to the body to maintain vitality, and the activity level depends on heart function^20^. EA at ST36 can produce a stronger effect than can EA at PC6 for heart function; this is consistent with the idea in TCM that the stomach envelopes food like the sea and provides the nutrients required by the five viscera and six bowels^21^.

In the sham, PC6, and ST36 groups, we identified 25 and 37 metabolites with significant fold changes in the heart and stomach tissues, respectively. The heatmap indicates that the heart tissue samples from the PC6 and sham groups exhibited similar patterns (Fig. 4A). However, the three groups exhibited three typical patterns for the stomach tissues (Fig. 4B). For the heart tissue samples, two main clusters (I: ST36; II: PC6 and sham) were identified using a dendrogram (Fig. 5A); for the stomach tissue samples, three main clusters (I-i: PC6; I-ii: sham; II: ST36) were identified (Fig. 5B). These results indicated that the effects of EA at ST36 on metabolites in both the heart and stomach tissues differed from those of EA at PC6 and the sham treatment. EA at PC6 and the sham treatment had similar effects on the metabolites in the rat heart tissues but different effects on the metabolites in the rat stomach tissues. This result indicates that specificity is a characteristic of acupoint treatment, although a given acupoint can be used to treat multiple diseases. For example, PC6 stimulation is performed to treat heart and gastric diseases; however, in TCM, this can also be achieved through ST36 stimulation^13,20^.

In the heart tissues, 10 metabolites were upregulated and 7 metabolites were downregulated by ST36 stimulation when compared with the sham treatment. These metabolites were distributed across 17 metabolism pathways, and 10 of them were specifically modulated by ST36 stimulation. In the heart tissues, five metabolites were upregulated and one metabolite was downregulated by PC6 stimulation when compared with the sham treatment. Only eight metabolism pathways were involved, and riboflavin metabolism responded independently to PC6 stimulation (Fig. 6). In the stomach tissues, 10 metabolites were upregulated and 18 metabolites were downregulated by ST36 stimulation when compared with the sham treatment, whereas 18 metabolites were upregulated and 5 metabolites were downregulated by PC6 stimulation when compared with the sham treatment. Each stimulation involved at least 28 metabolism pathways, but only 6 were affected by both ST36 and PC6 stimulation (Fig. 7). In addition, at least 20 metabolic pathways in the heart tissues were reversely modulated by ST36 and PC6 stimulation; at least 32 metabolite pathways in the stomach tissues were reversely modulated by ST36 and PC6 stimulation (Fig. 8). The ST36 and PC6 groups used different pathways. These results indicate that EA at ST36 and PC6 has different effects on metabolism in the heart and stomach tissues. The metabolic reprograming resulting from EA at ST36 or PC6 may directly or indirectly modify the function of these organs to create therapeutic indications and efficacies.

Several questions remain unanswered. First, no imaging study has been conducted to identify the location or pathway of action from EA at a given acupoint to a target organ. Second, although EA at a given acupoint can affect organ function through multiple metabolic pathways, the correlated effects remain poorly understood. Third, EA at a given acupoint also affects neurotransmitter levels in the brain, and the route of signal transmission from an acupoint to an organ requires clarification.

EA-generated signals can target the heart and stomach as well as the brain for local modulation via the sham, ST36, and PC6 acupoints. EA stimulation at ST36 may cause a more significant modulation of metabolism to the heart by affecting pathways different from those affected by PC6 stimulation. This study also supported the TCM theory that the pericardium meridian connects the heart and the stomach and that the stomach is the source of energy for internal organs.

## Methods

### Animals

Male Sprague–Dawley (SD) rats weighing 200–300 g were purchased from Bio-LASCO Taiwan and raised at the animal center of China Medical University (CMU) in a 12:12 h light–dark cycle environment. Room temperature was set to 20–24 °C, and humidity was controlled to 50–70% by using an air conditioner. The rats were free fed and provided sufficient drinking water and food. The laboratory use of animals was approved by the Institutional Animal Care and Use Committee of CMU (study on the relationship among acupoints, the brain, and visceral organs; CMUIACUC-2017-349) and implemented in accordance with the *Guide for the Use of Laboratory Animals* (National Academy Press). The study was also conducted in accordance with the Animal Research: Reporting of In Vivo Experiments guidelines.

### Animal models

A total of 18 SD rats were randomly divided into three groups (six rats in each group) as per the protocol used in our previous study^1^. (1) In the sham group, the rats were anesthetized with 2% isoflurane, and four acupuncture needles were subcutaneously inserted into their bilateral ST36 (as a cathode) and Shangjuxu (ST37) acupoints (as an anode); these acupoints share characteristics with those found in humans. Thereafter, the needles were connected to a Trio 300 electrical stimulator (Grand Medical Instrument Co., Ito, Japan) and paired ipsilaterally, but no electrical stimulation was administered. This procedure was performed for 20 min a day for 5 consecutive days. (2) In the ST36 group, the procedure was almost identical to that applied to the sham group, with the difference being that the acupuncture needles were inserted intramuscularly at ST36 as a cathode and at ST37 as an anode and that 2-Hz EA was administered. The intensity of electrical stimulation was intended to induce slightly visible muscle twitching. (3) In the PC6 group, the procedure was almost identical to that applied to the ST36 group, with the difference being that the acupuncture needles were inserted at the bilateral PC6 as a cathode and at the Jianshi acupoints as an anode. Subsequently, the rats were administered 3–5% isoflurane anesthesia, and their heart apex and stomach fundus were collected for metabolomic analysis.

### Metabolite sample preparation

The rat tissue samples were processed by following the procedures used in our previous studies^1,22^. The heart and stomach tissues were homogenized using 1-mm zirconium oxide grinding beads at a ratio of 1 mg of tissue to 10 μL of ultrapure water and then centrifuged at 14,000 rpm for 10 min. Each supernatant was collected as an aliquot of 100 μL and mixed with 300 μL of 100% methanol. After 14,000-rpm centrifugation for 10 min, 150 μL of supernatant (obtained using methanol extraction) was transferred to a new microtube and vacuum dried. Each dry metabolite sample was reconstituted in 50 μL of ultrapure water and subjected to 14,000-rpm centrifugation for 10 min. The supernatant was then transferred to an insert vial and kept in an autosampler at 10 °C for sample injection.

### Liquid chromatography-mass spectrometry

Metabolite samples were analyzed by applying the conditions of our previously established methods^1,22^. The LC–MS system consisted of an ultraperformance LC system (ACQUITY UPLC I-Class, Waters), and an electrospray ionization (ESI)/atmospheric pressure chemical ionization source of 4 kDa quadrupole time-of-flight mass spectrometer (Waters VION, Waters). A BEH C18 column (2.1 × 100 mm^2^, Walters) was used to perform chromatographic separation. The flow rate was set to 0.2 mL/min, with a column temperature of 35 °C, and each sample was subjected to LC–MS with a 5-μL sample injection. The sample contents were eluted using 99% mobile phase A (ultrapure water + 0.1% formic acid) and 99% mobile phase B (100% methanol + 0.1% formic acid); subsequently, the contents were held at 1% B for 0.5 min, raised to 90% B for 5.5 min, held at 90% B for 1 min, and then lowered to 1% B for 1 min. The column was equilibrated by pumping 1% B for 4 min. An LC–MS chromatogram was acquired using ESI + and ESI − modes under the following conditions: capillary voltage of 2.5 kV, source temperature of 100 °C, desolvation temperature of 250 °C, cone gas maintained at 10 L/h, desolvation gas maintained at 600 L/h, and acquisition in MS^E^ mode with a range of 100–1000 m/z and a 0.5-s scan time.

### Data processing

The profiling data were acquired using UNIFI software (Waters) and processed using Progenesis QI software with EZinfo (Waters). The LC–MS signals acquired in ESI+ and ESI− modes were uploaded to Progenesis QI with EZinfo for data normalization, peak picking, compound measurement, and statistical analysis. The LC–MS signals were then converted to datasets by using Progenesis QI. The datasets were subjected to statistical and pathway analysis by applying the MetaboAnalysis 5.0 tool (https://www.metaboanalyst.ca//faces/ModuleView.xhtml).

### Ethics statement

The protocol was approved by the Animal Care and Use Committee of China Medical University (study on the relationship among acupoints, the brain, and visceral organs; CMUIACUC-2017-349). The study is also reported in accordance with ARRIVE guidelines.

### Consent for publication

This study did not include any human data.

## Data Availability

Please contact the author for data requests.
